# Emergency entity relationship extraction for water diversion project based on pre-trained model and multi-featured graph convolutional network

**DOI:** 10.1371/journal.pone.0292004

**Published:** 2023-10-09

**Authors:** Li Hu Wang, Xue Mei Liu, Yang Liu, Hai Rui Li, Jia QI Liu, Li Bo Yang

**Affiliations:** 1 School of Management and Economics, North China University of Water Resources and Electric Power, Zhengzhou, Henan, 450046, China; 2 School of Information Engineering, North China University of Water Resources and Electric Power, Zhengzhou, Henan, 450046, China; 3 Collaborative Innovation Centre for Efficient Utilization of Water Resources, North China University of Water Resources and Electric Power, Zhengzhou, Henan, 450046, China; National University of Sciences and Technology NUST, PAKISTAN

## Abstract

Using information technology to extract emergency decision-making knowledge from emergency plan documents is an essential means to enhance the efficiency and capacity of emergency management. To address the problems of numerous terminologies and complex relationships faced by emergency knowledge extraction of water diversion project, a multi-feature graph convolutional network (PTM-MFGCN) based on pre-trained model is proposed. Initially, through the utilization of random masking of domain-specific terminologies during pre-training, the model’s comprehension of the meaning and application of such terminologies within specific fields is enhanced, thereby augmenting the network’s proficiency in extracting professional terminologies. Furthermore, by introducing a multi-feature adjacency matrix to capture a broader range of neighboring node information, thereby enhancing the network’s ability to handle complex relationships. Lastly, we utilize the PTM-MFGCN to achieve the extraction of emergency entity relationships in water diversion project, thus constructing a knowledge graph for water diversion emergency management. The experimental results demonstrate that PTM-MFGCN exhibits improvements of 2.84% in accuracy, 4.87% in recall, and 5.18% in F1 score, compared to the baseline model. Relevant studies can effectively enhance the efficiency and capability of emergency management, mitigating the impact of unforeseen events on engineering safety.

## Introduction

The emergency plan for water diversion project plays a significant role in guiding the efficient and orderly implementation of emergency management efforts [1]. With the advancement of water conservancy informatization, the field of emergency management for water diversion project has accumulated a vast array of document-based emergency plans. These plans exhibit characteristics such as significant scale, diverse structures, and an abundance of professional terminology [2,3]. Currently, emergency decision-makers are still required to manually peruse and search for document-based emergency plans when formulating emergency strategies for unforeseen events. This reliance on a traditional, labor-intensive approach not only significantly impairs the efficiency of emergency decision-making but also hinders the full utilization of historical experiences and real-time information [4–6]. Hence, the extraction of valuable emergency decision-making knowledge from vast unstructured or semi-structured data becomes crucial in enhancing the efficiency and capacity of emergency management.

Conventional methods of knowledge extraction heavily rely on rule-matching techniques [7–9], which involve extracting structured knowledge from unstructured or semi-structured texts using predefined sets of rules [10,11]. For instance, literature [12] presents a city fire knowledge extraction method based on semantic rules. In literature [13], a knowledge extraction method based on semantic information and syntactic features is proposed for the field of urban traffic emergency response. In the literature [14], a network security named entity recognition model has been developed based on regular expressions and dictionary matching. Rule-based knowledge extraction methods possess advantages such as strong interpretability and high controllability, but they suffer from drawbacks such as cumbersome rule definition, poor scalability, and low extraction accuracy [15,16].

Deep learning methods based on recurrent neural networks (RNN) can effectively leverage the temporal dependencies in text and better capture the complex relationships within the data, thus alleviating the drawbacks and limitations of rule-based approaches [17–19]. In the literature [20], an attention-based bidirectional long short-term memory network model (ATT-BILSTM) is posited, serving the purpose of discerning network emergency events. In the literature [21], a knowledge extraction method focusing on industrial health management is proposed, which combines BILSTM with conditional random field (CRF). In the literature [22], the BILSTM model has been augmented into a bidirectional gated recurrent unit network (BIGRU), proposing a method for extracting emergency knowledge related to power accidents known as BIGRU-CRF. The knowledge extraction methods based on RNN take into account the temporal dependency of the text. However, emergency plan texts also exhibit strong spatial dependency, and mining solely from the temporal dimension has certain limitations [23,24].

Due to the spatial dependency-capturing capability of convolutional neural networks (CNN), scholars have proposed several knowledge extraction methods based on CNN [25,26]. Literature [27] constructs a knowledge graph for emergency decision-making in the power grid domain using TextCNN. In literature [28], an advanced approach is presented, delineating a vehicular equipment malfunction entity recognition method that is founded upon the integration of CNN and CRF. Since knowledge graphs inherently represent graph-structured data, researchers have attempted to introduce graph neural networks (GNN) in order to address the challenge of complex relationship mining in non-Euclidean data [29,30]. The literature [31] utilizes the graph convolutional network (GCN) model to extract critical knowledge within the realm of oil and gas pipeline emergencies. In the literature [32], a method for extracting urban traffic accident information based on GCN is proposed to tackle the issue of conventional deep learning models’ inability to capture graph structural features. The GCN method excels in capturing the spatial dependencies of graph data, yet when faced with long textual data, the predictive errors of the GCN model accumulate, resulting in a decrease in extraction accuracy [33,34].

With the emergence of advanced pre-training models such as bidirectional encoder representation from transformers (BERT) [35], generative pre-trained transformer (GPT) [36], and text-to-text transfer transformer (T5) [37], researchers have endeavored to leverage these models to compensate for the limitations of CNN [38–40]. In the literature [41], the amalgamation of BERT and CNN models gives birth to an innovative methodology for extracting knowledge related to fire emergencies, referred to as the BERT-CNN approach. In the literature [42], a method based on BERT-RNN for identifying emergency entities for earthquake disaster is proposed. In the literature [43], an integration of BERT, BILSTM, and CRF is employed for the purpose of nested named entity recognition in the field of geological disasters. Although the aforementioned studies have achieved promising results in their respective fields, the focus during the pre-training stage primarily revolves around traditional pre-training tasks [44]. In addition, regarding the research on GCN, it is common to only consider the relationships of single-feature neighboring nodes, thereby overlooking the broader information from neighboring nodes [45,46].

Based on the above discussion, this study proposes a multi-feature GCN method (PTM-MFGCN) based on a pre-trained model to solve the problems of numerous technical terms and complex relationships faced by emergency knowledge extraction of water diversion project. The main contributions are as follows:

- By employing the technique of random masking on specialized terminology, the model acquires a deeper comprehension of the meaning and application of such terms within specific domains. Consequently, this elevates the network’s proficiency in extracting and recognizing professional terminology;
- By incorporating a multi-feature adjacency matrix, the network can effectively capture a broader range of information from neighboring nodes, thus enhancing its ability to process complex relationships;
- Extraction of emergency entity relationships for water diversion project based on PTM-MFGCN and construction of emergency knowledge graph for water diversion project;

The remaining sections of this paper are organized as follows: Section 2, describes the progress of work related to PTM-MFGCN. Sections 3 and 4 detail the construction process of PTM-MFGCN and the process of entity relationship extraction. Section 5 presents the experimental protocol, results and discussion. Finally, section 6 concludes the paper and indicates future research directions.

## Related work

### Pre-trained language models

Pretrained language models aim to acquire extensive linguistic knowledge through self-supervised learning using text corpora [47]. Early iterations of pre-trained language models predominantly relied on n-gram and rule-based approaches. In the literature [48], the Global Vectors model was proposed, which accomplishes pre-training by incorporating both word co-occurrence statistics and global semantic information. In the literature [49], the Word Vector model was proposed, which achieves pre-training by representing words as continuous vectors through self-supervised learning.

With the advancement of deep learning, the literature [50] introduced the Transformer model, which employs self-attention mechanisms for sequence modeling. The Transformer model effectively captures long-range dependencies in text through its self-attention mechanism, leading to significant performance improvements in machine translation tasks. Subsequently, the literature [35] introduced the BERT model, building upon the foundation of Transformer. The BERT model employs a strategy of pre-training and fine-tuning, conducting extensive pre-training on large-scale unsupervised data and subsequently fine-tuning on downstream tasks. The groundbreaking innovation of the BERT model has yielded exceptional results across multiple natural language processing tasks, sparking widespread scholarly attention towards pre-trained language models.

### GNN

GNN, as a deep learning model, is designed for handling graph data [51]. Early research on GNN primarily focused on the extraction of graph features and the learning of representations. The paper [52] introduced the graph recursive neural network (GRN), which employs iterative propagation of node features to learn node representations.

However, the GRN primarily relies on the information from local neighboring nodes and fails to effectively capture global graph structural information. The literature [53] introduced GCN, which extends convolutional operations to the field of graphs, updating node representations by aggregating information from neighboring nodes. The introduction of GCN bridged the gap in graph domain’s representation learning, laying the foundation for subsequent research. The literature [54] introduced the graph attention network (GAT) building upon GCN, incorporating attention mechanisms to model the weights between nodes. GAT possesses the capability to dynamically allocate attention weights among different nodes, enabling more precise aggregation of neighboring node information.The introduction of GAT further enhances the modeling capacity for complex graph structures.

## PTM-MFGCN entity relationship extraction model

### Pre-training mechanism

The emergency plan for water diversion project involves a wide range of specialized terminology. By employing pre-trained language models, it is possible to transfer the linguistic knowledge acquired during the pre-training phase to the task of extracting emergency knowledge. During the pre-training phase PTM-MFGCN enables the model to better understand the meaning and usage of terminology in a particular domain by randomly masking the terminology.

**Fig 1:**
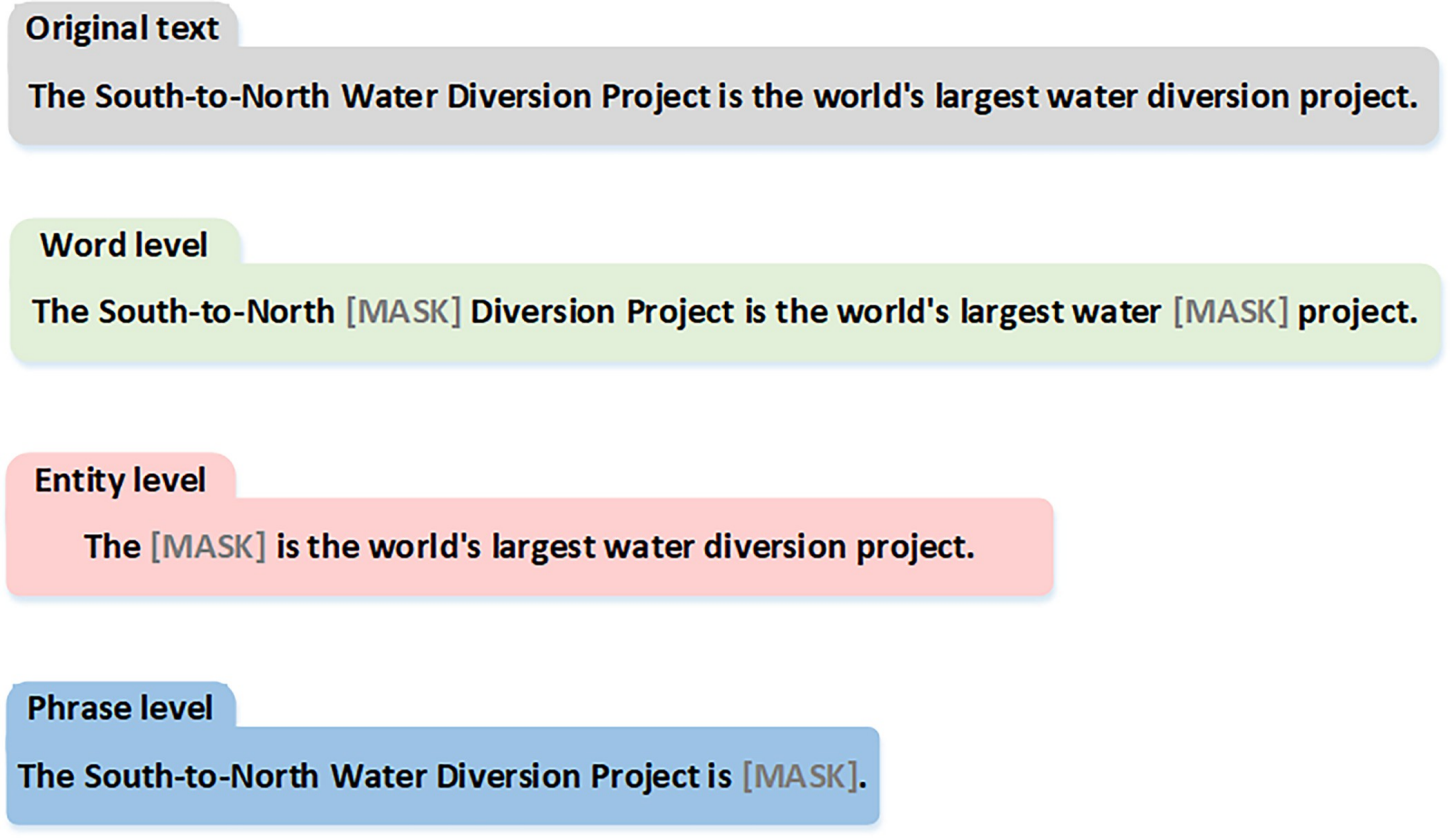
The process by which specialized terms are randomized MASK in the pre-training phase.

Terminology masking allows the model to learn the contextual features of terms by predicting the masked content by randomly masking different technical terms. Specifically, by applying three levels of masking operations (character-level, entity-level, and phrase-level) to the input text. In the character-level masking, 15% of the domain-specific terms are randomly selected for masking. The selected terms have an 80% probability of being replaced with "[MASK]", a 10% probability of being replaced with other terms, and a 10% probability of remaining unchanged. The character-level masking strategy enables the model to learn basic word representations, but it struggles to capture advanced semantic knowledge. Entity-level masking begins by analyzing the specialized term entities in the sentence, and subsequently applies random masking to these entities to uncover semantic knowledge that encompasses the terms. Phrase-level masking initially employs a sentence segmentation tool to extract phrases from the text, followed by the random selection of a subset of these phrases for masking operations, thus facilitating the modeling of phrase-level semantic knowledge. Fig 1 illustrates the process of randomly masking professional terms during the pre-training phase.

### MFGCN construction

GCN is an extension of CNN in non-Euclidean spaces [55]. GCN employs convolutional operations to encode local information and learns more global information through the message passing of multiple GCN layers. Given a sentence consisting of n words, it is common to construct an adjacency matrix, represented as *AϵR*^n*n^, using syntactic dependency trees [56], to represent the sentence graph. The value of *A*_*ij*_ indicates whether the *i*rd node is connected to the *j*rd node. Specifically, if the *i*rd node is connected to the *j*rd node, then *A*_*ij*_ = 1; otherwise, *A*_*ij*_ = 0. All nodes in the graph are associated with a label, and GCN utilizes the known label information of nodes to predict the labels of unknown nodes for classification. For the *L*rd node in the *i*rd layer, the hidden representation is denoted as $h_{i}^{l}$, and its calculation is expressed by the following formula:

$$h_{i}^{l}=\sigma \left(\sum_{j=1}^{n}A_{ij}W^{l}h_{j}^{l-1}+b^{l}\right)$$ (1)

where σ denotes the activation function, *W* represents the weight matrix, *b* represents the bias term.

**Fig 2:**
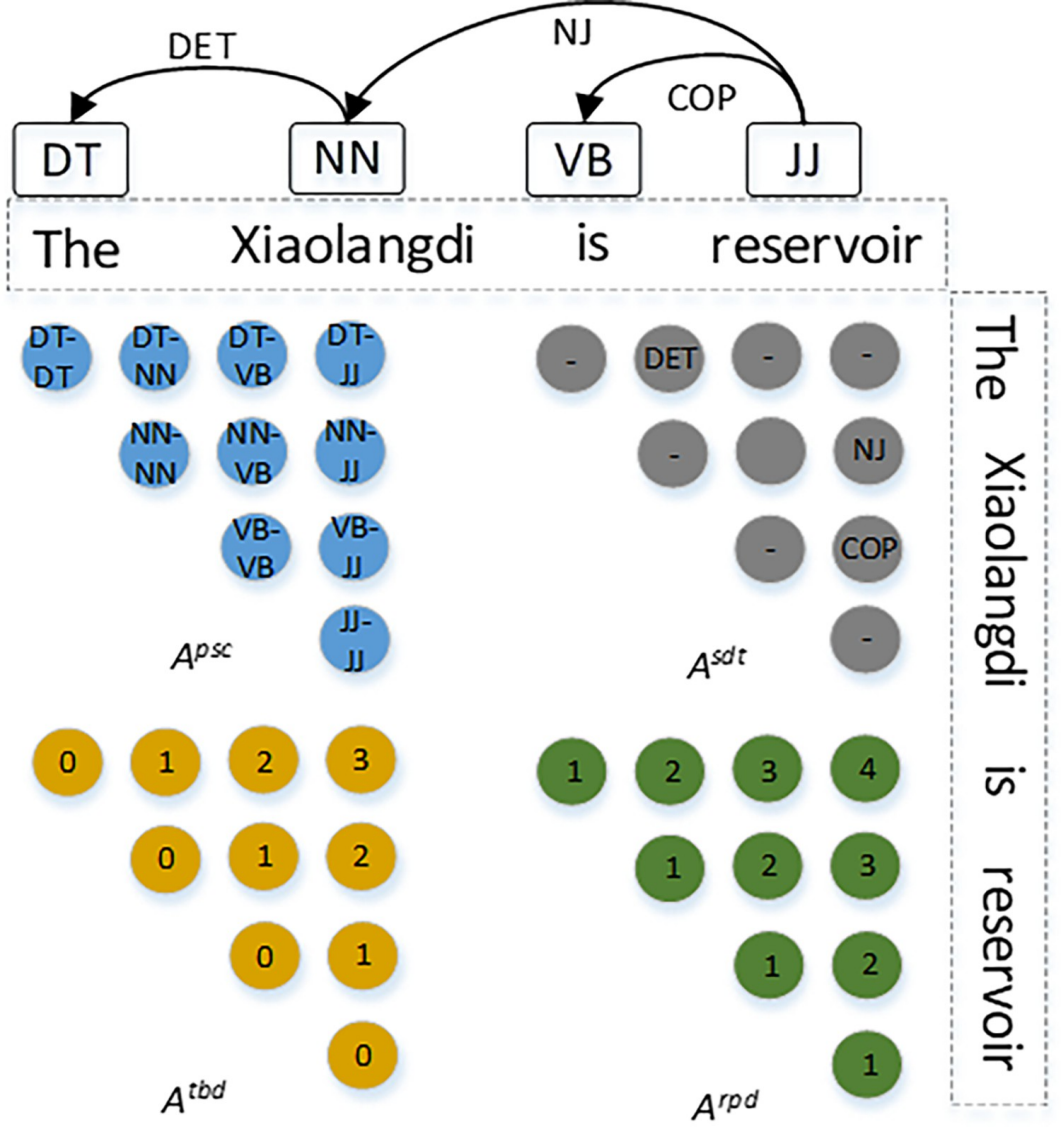
Construction logic of the four MFGCN adjacency matrices (DT, NN, VB and JJ for pronouns, nouns, verbs and adjectives; DET, NJ and COP for accusative relations, noun subjects and juxtapositions).

This study introduces four types of linguistic features, which are used to initialize four adjacency matrices based on these features. The multi-feature adjacency matrix is composed of the part-of-speech combination vector *A*^*psc*^, the syntactic dependency vector *A*^*sdt*^, the tree-based distance vector *A*^*tbd*^, and the position vector *A*^*rpd*^, where the definitions of *A*^*psc*^, *A*^*sdt*^, *A*^*tbd*^, *A*^*rpd*^ are illustrated in Fig 2. Based on the four adjacency matrices, the local information is encoded through convolutional operations, resulting in the vector representations of hidden nodes *H*^*psc*^, *H*^*sdt*^, *H*^*tbd*^, and *H*^*rpd*^. The pooling function and the join operation are applied to all hidden layer nodes to obtain the model output, as defined in the following equation.


$$H=\tau_{pool}(H^{psc}⊕H^{sdt}⊕H^{tbd}⊕H^{rpd})$$ (2)

Among them, *τ*_*pool*_ denotes the pooling function, and ⊕ serves as the connectivity operator.

### PTM-MFGCN model construction

**Fig 3:**
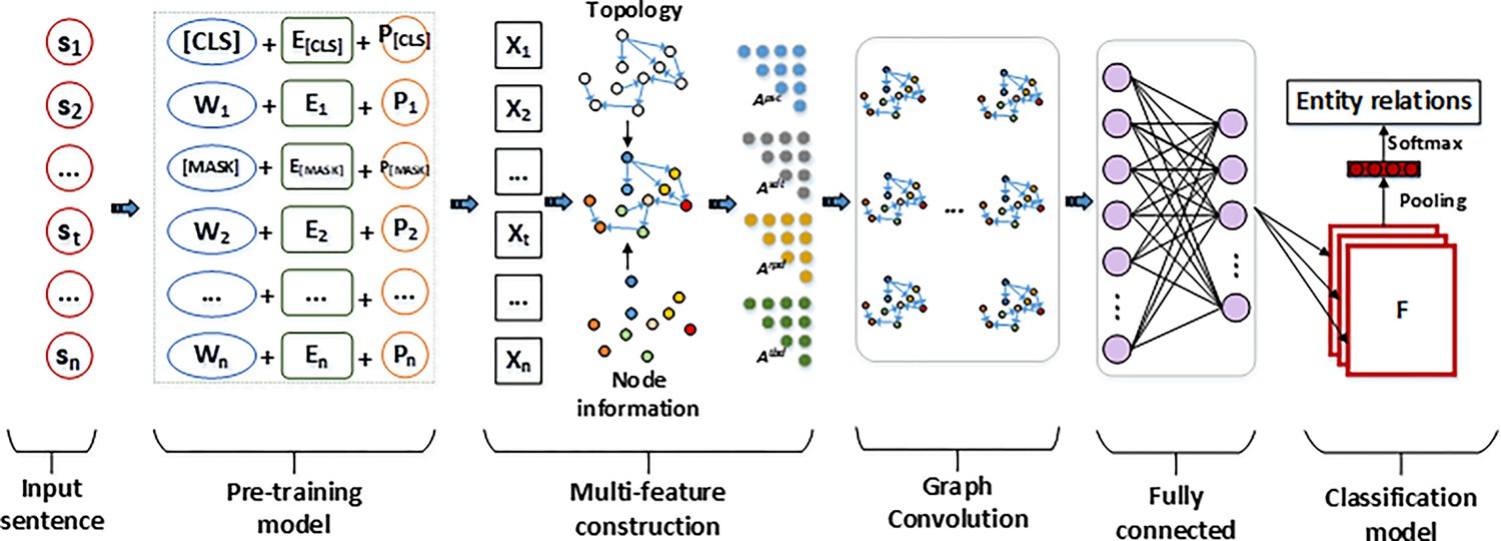
PTM-MFGCN model data processing flow.

PTM-MFGCN comprises two main modules: The pre-training module and the graph convolution module. The pre-training module enhances the network’s ability to extract domain-specific terminology, while the graph convolution computation enhances the network’s capability to handle complex relationships. Fig 3 illustrates the fundamental workflow of the PTM-MFGCN model, which is implemented as follows.

Pre-training module: Represent the text sequence of length *n* as *S* = [*s*_1_, *s*_1_,…*s*_*n*_], and prepend the marker "[CLS]" at the beginning of the sequence as a classification flag. The [CLS] symbol serves as an encoded representation that signifies the entirety of the input sequence, enabling the model to proficiently undertake classification and other downstream tasks. Using character-level, entity-level, and text-level encoding masking mechanisms, conceal specialized terminologies within the sentence. The inputs to the pre-trained model are defined as follows.


$$X=f_{pre}\left([[CLS],w_{1},[MASK],\ldots ,w_{n}[⊕[E_{\left[CLS\right]},e_{1},E_{\left[MASK\right]},\ldots ,e_{n}]⊕[p_{\left[CLS\right]},p_{1},p_{\left[MASK\right]},\ldots ,p_{n}]\right)$$ (3)

Among them, [*MASK*] denotes the masked term characters, *E*_[*MASK*]_ denotes the masked term entities, *p*_[*MASK*]_ represents the masked term context, and *X* denotes the word vectors extracted using the pre-training model.

Graph convolution computation module: The pre-training module produces a spatial vector representation of the sentence. Based on part-of-speech combination vectors, syntactic dependency vectors, tree distance vectors, and positional vectors, we construct multi-feature adjacency matrices *A*^*psc*^, *A*^*std*^, *A*^*tbd*^, and *A*^*rpd*^. Taking *A*^*psc*^ as an example, feature extraction is performed using graph convolution and is defined as follows.


$$H^{psc}=\sigma \left(\sum_{j=1}^{n}A^{psc}Wh+b\right)$$ (4)

Finally, the extracted features are utilized as inputs for the pooling function and the classification function to carry out entity relation extraction. For model training, the cross-entropy function is employed as the loss function, defined as follows.


$$\tau =-\frac{1}{N}\sum_{i=1}^{n}y^{\left(i\right)}*log({\hat{y}}^{\left(i\right)})+(1-y^{\left(i\right)})*log(1-{\hat{y}}^{\left(i\right)})$$ (5)

Among them, ${\hat{y}}^{\left(i\right)}$ denotes the output of the model, *y*^(*i*)^ denotes the true labels, and *N* denotes the number of entity triples.

## Emergency entity relationship extraction based on PTM-MFGCN

**Fig 4:**
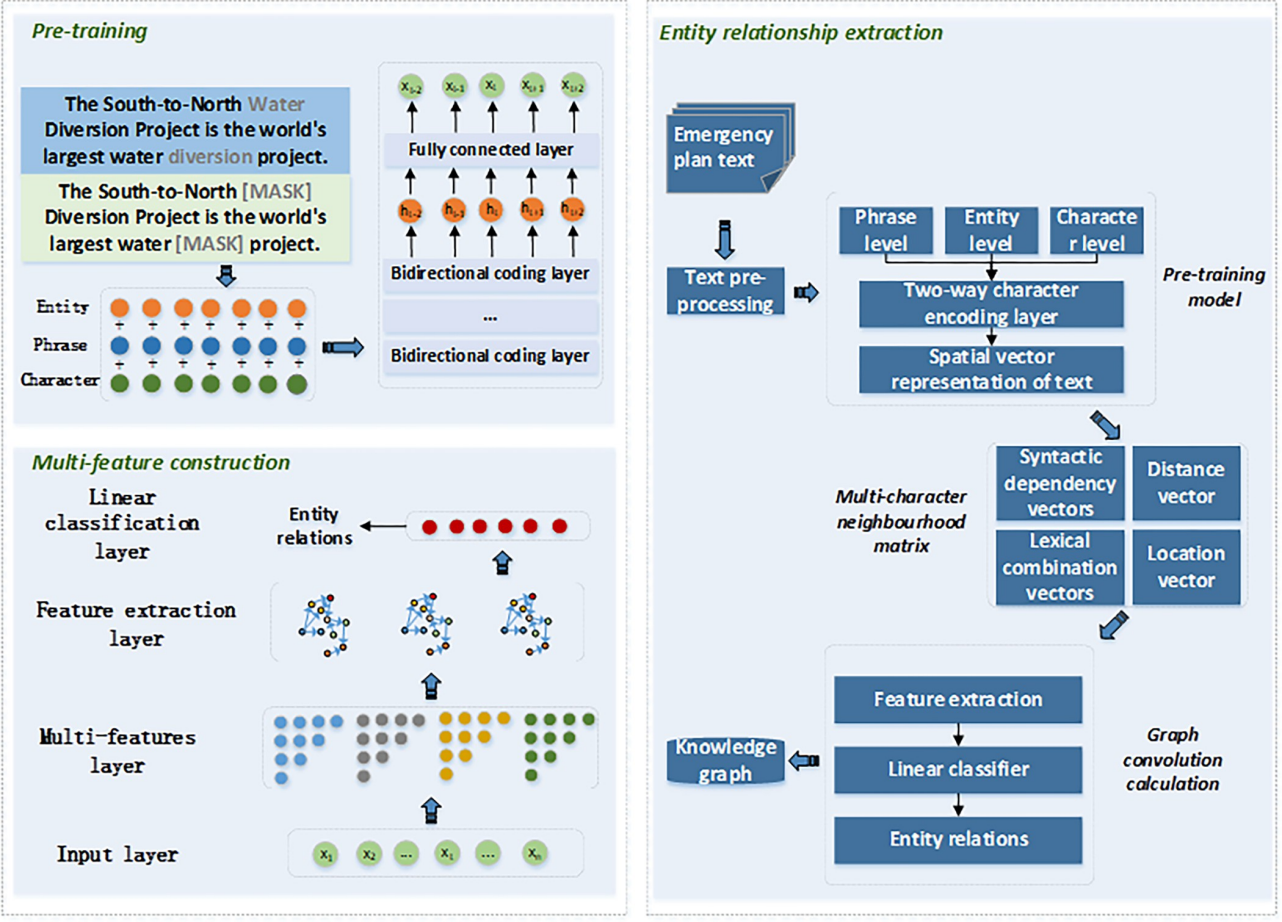
Emergency entity relationship extraction for water diversion project based on PTM-MFGCN.

The study focuses on the comprehensive emergency plans at the provincial and municipal levels, as well as 17 specific emergency plans released between 2014 and 2021 for the South-to-North Water Diversion Project in China. The South-to-North Water Diversion Project in China is a large-scale inter-basin water transfer project implemented to alleviate water scarcity issues in the northern region of China. Since its comprehensive implementation, the project has conveyed a volume of 58.6 billion cubic meters of water, effectively alleviating water scarcity issues in the northern region of China. Based on the PTM-MFGCN, the emergency entity relationship extraction for water diversion projects primarily consists of three stages: Text preprocessing, entity relationship extraction, and storage of knowledge graph triplets. The basic disposal process of the method is given in Fig 4 and the procedure is as follows.

1. Text preprocessing: Initially, employing character-level, entity-level, and phrase-level masking strategies, random masking is applied to professional terminology within the original emergency plan text, followed by the addition of a classification symbol [CLS] at the beginning of the text sequence.
2. Entity relation extraction: By employing pre-training tasks masked with random terminology, contextual features and semantic information of specialized terminology are extracted. The output of the pre-training model corresponds to the spatial vector representation of the sentence, denoted as *X* = [*x*_1_, *x*_1_,…*x*_*n*_]. Based on *X*, a multi-feature adjacency matrix is constructed, encompassing *A*^*psc*^, *A*^*std*^, *A*^*tbd*^, and *A*^*rpd*^. The spatial vector *X* obtained during the pre-training phase is combined with the multi-feature adjacency matrix as the input for the GCN model to facilitate feature extraction. Finally, the features extracted by the GCN model are used as inputs to the classification model for entity extraction and relation extraction.
3. Knowledge graph triplet storage: After named entity recognition and relation extraction, the emergency data of the water diversion project has been transformed from unstructured textual data into structured knowledge triplets. The Neo4j graph database is employed for knowledge storage, where the acquired structured knowledge is stored in a semantic network, primarily composed of nodes and edges.

## Experiment

Entity relation extraction serves as the foundation for constructing a knowledge graph. In this section, we validate the effectiveness of PTM-MFGCN through the task of entity relation extraction in the context of emergency plans for water diversion project. Firstly, an explanation will be provided regarding the datasets, baseline models, and evaluation indexes used in the experiment. Subsequently, the experiment will be divided into three groups to achieve distinct research objectives.

1. An experiment will be conducted to extract entity relationships using the PTM-MFGCN model on the emergency plan data of the water diversion project. The experimental results will be compared and analyzed with those of previous state-of-the-art models to assess the effectiveness of the PTM-MFGCN model;
2. Conducting a sensitivity analysis experiment for hyperparameters to verify the model’s sensitivity to the settings of hyperparameters and evaluate its performance;
3. The impact of term mask-based pre-training tasks with multi-feature graph convolution on the performance of PTM-MFGCN is analysed through ablation experiments.
4. Based on actual emergency scenarios, an evaluation of the emergency knowledge graph constructed in this study is conducted through case retrieval. This assessment determines the efficacy of the method proposed in this paper in accurately acquiring pertinent information to underpin emergency decision-making.

### Data set and baseline model

This study utilizes the comprehensive emergency plans at the provincial and municipal levels, as well as 17 specific emergency plans released from 2014 to 2021, pertaining to the South-to-North Water Diversion Project in China, as the experimental data. The emergency plans contain a total of 790,000 entity relations triad of four phases: forecast and warning, graded response, emergency disposal and post-security. Table 1 provides some examples of emergency plans. The emergency plan data is divided into training and testing sets, with a partition ratio of 7:3. The baseline models selected for this study are widely used named entity recognition models and relation extraction models. The baseline models include BILSTM [21], FastText [23], TextCNN [27], BERT-CNN [41], and BERT-BILSTM-CRF [43,44]. Table 2 presents the parameter configurations for each model.

**Table 1:** Detailed example of the dataset.

Emergency plan	Example of text content	Emergency scenarios
Emergency plan for major floods in the South-to-North Water Diversion Project	Risk classification: According to the severity and scope of the possible damage caused by major floods, floods are classified into four levels, from high to low, as level Ⅰ, Ⅱ, Ⅲ, Ⅳ.	Flood disaster
Emergency plan for earthquake disaster in the South-to-North Water Diversion Project	Risk analysis: When an earthquake occurs, it may cause damage to the project. The risks associated with earthquakes fall into three main categories, structural damage to the project, loss of control of the automated dispatch system, and water quality contamination.	Earthquake disaster
Emergency plan for fire accidents in the South-to-North Water Diversion Project	Emergency disposal: Urgently contact the fire, medical and other emergency rescue departments in the place of the incident, and report the accident to the superior.	Fire incident

**Table 2:** Values of the model parameters.

Hyperparameters	Description	Values
Hidden layer dimension	Number of hidden layer neurons in the network.	32
Embedding dimension	Vector dimensionality when converting text data to vector representation.	200
Dropout rate	The proportion or probability of randomly discarding neurons.	0.2
Epochs	Number of model training times.	500

### Evaluation indexes

For model evaluation, this study employs accuracy, recall, and F1 score as the evaluation indexes. Accuracy refers to the proportion of correctly predicted samples by a model out of the total number of samples. It measures the overall predictive accuracy of the model. Recall is the proportion of samples correctly predicted as positive by the model out of the total number of true positive samples. It measures the model’s ability to identify positive instances. The F1 score is the harmonic mean of accuracy and recall, used to evaluate the overall performance of a model. The higher the values of accuracy, recall, and F1 score, the better the performance of the model. Accuracy (P), recall (R), and F1 score are defined as follows:

$$P=\frac{T_{P}}{T_{P}+F_{P}}100\%$$ (6)

$$R=\frac{T_{P}}{T_{P}+F_{N}}100\%$$ (7)

$$F1=\frac{2P∙R}{P+R}100\%$$ (8)

Among them, *T*_*P*_ denotes the number of correctly predicted entities, *F*_*P*_ denotes the number of predicted entities that are not actual entities, and *F*_*N*_ denotes the number of entities that were not predicted. Furthermore, this study integrates the actual emergency scenarios of engineering, employing knowledge usability as a qualitative assessment index to appraise the emergency knowledge graph formulated in this paper. This assessment serves to ascertain the capability of the method proposed in this study to precisely acquire valuable information to bolster emergency decision-making.

### Analysis of entity relationship extraction results

**Fig 5:**
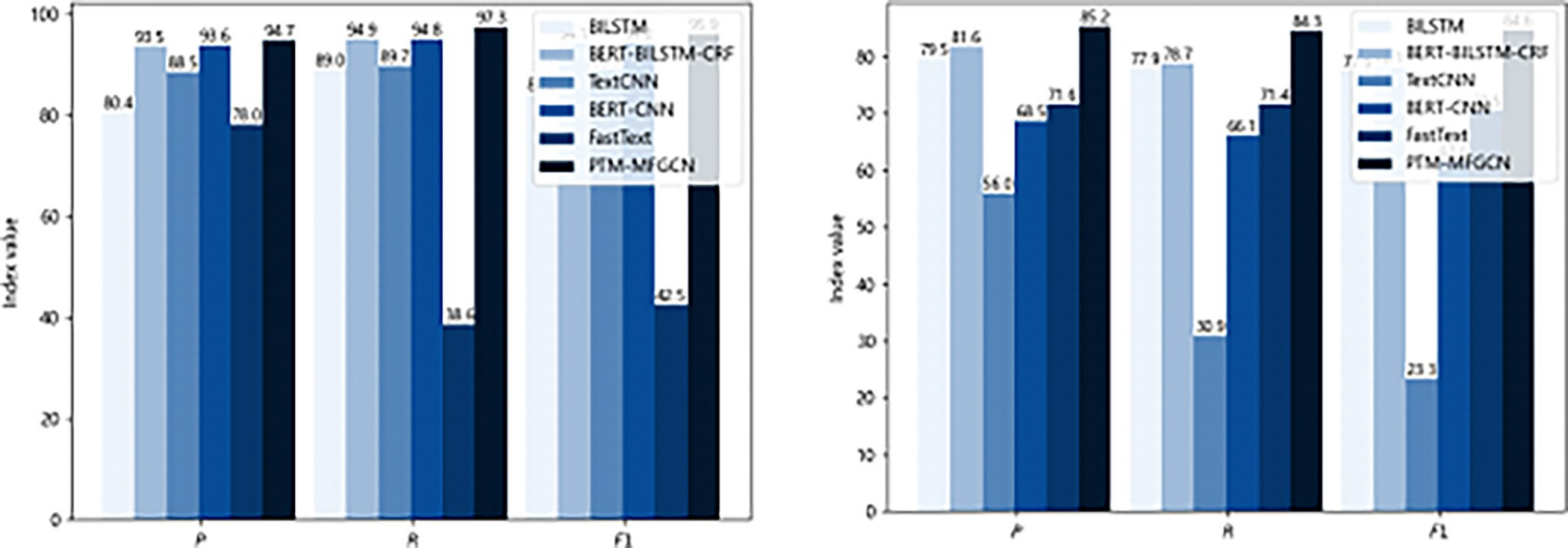
Comparison of performance levels by model.

Fig 5 and Table 3 present the experimental results of different models. In terms of entity extraction, FastText performed the poorest with precision, recall, and F1 values of 78.03, 38.60, and 42.52, respectively. FastText utilizes character-level n-gram features to represent text, which limits its ability to comprehend complex semantics. The BERT-BILSTM-CRF performed better with an accuracy recall and F1 of 93.49, 94.87 and 94.09 respectively. Based on pre-training and a bidirectional language model, BERT-BILSTM-CRF possesses the capability to delve deep into the intricate relationships between vocabulary, syntax, and semantics, thereby providing a comprehensive contextual representation. The PTM-MFGCN model proposed in this paper achieves an accuracy, recall, and F1 score of 94.65, 97.28, and 95.94, respectively. Compared to BERT-BILSTM-CRF, the PTM-MFGCN model achieves an improvement of 1.24% in accuracy, 2.54% in recall, and 1.97% in F1 score. In the field of emergency knowledge extraction for water diversion projects, there is a plethora of specialized terminology within the emergency plans for water diversion projects. Due to the inability of traditional pre-training models to perform targeted pre-training, BERT-BILSTM-CRF exhibits weaker extraction capabilities for emergency knowledge in water diversion projects compared to PTM-MFGCN. In terms of relation extraction, BERT-BILSTM-CRF achieves accuracy, recall, and F1 scores of 81.56%, 78.67%, and 78.06% respectively, while PTM-MFGCN achieves accuracy, recall, and F1 scores of 85.18%, 84.33%, and 84.60% respectively. Compared to BERT-BILSTM-CRF, PTM-MFGCN achieves an improvement of 4.43% in accuracy, 7.19% in recall, and 8.38% in F1 score. The emergency plan for water diversion projects exhibits characteristics such as complex relationship. The proposed graph convolution-based relation extraction model in this paper demonstrates superior performance in handling complex graph-structured data, thereby enhancing its capability in the domain. In general, the PTM-MFGCN proposed in this paper surpasses widely employed state-of-the-art models in terms of entity relation extraction results. This indicates the capability of PTM-MFGCN to effectively accomplish the task of emergency entity relation extraction for water diversion project.

**Table 3:** Evaluation index values for each model.

Models	Entity extraction			Relationship extraction		
	*P*	*R*	*F1*	*P*	*R*	*F1*
**BILSTM**	80.37	88.98	84.17	79.50	77.90	77.46
**BERT-BILSTM-CRF**	93.49	94.87	94.09	81.56	78.67	78.06
**TextCNN**	88.46	89.66	88.93	55.98	30.90	23.33
**BERT-CNN**	93.60	94.81	94.19	68.51	66.14	61.60
**FastText**	78.03	38.60	42.52	71.56	71.43	70.55
**PTM-MFGCN**	94.65	97.28	95.94	85.18	84.33	84.60

### Hyperparametric sensitivity analysis

**Fig 6:**
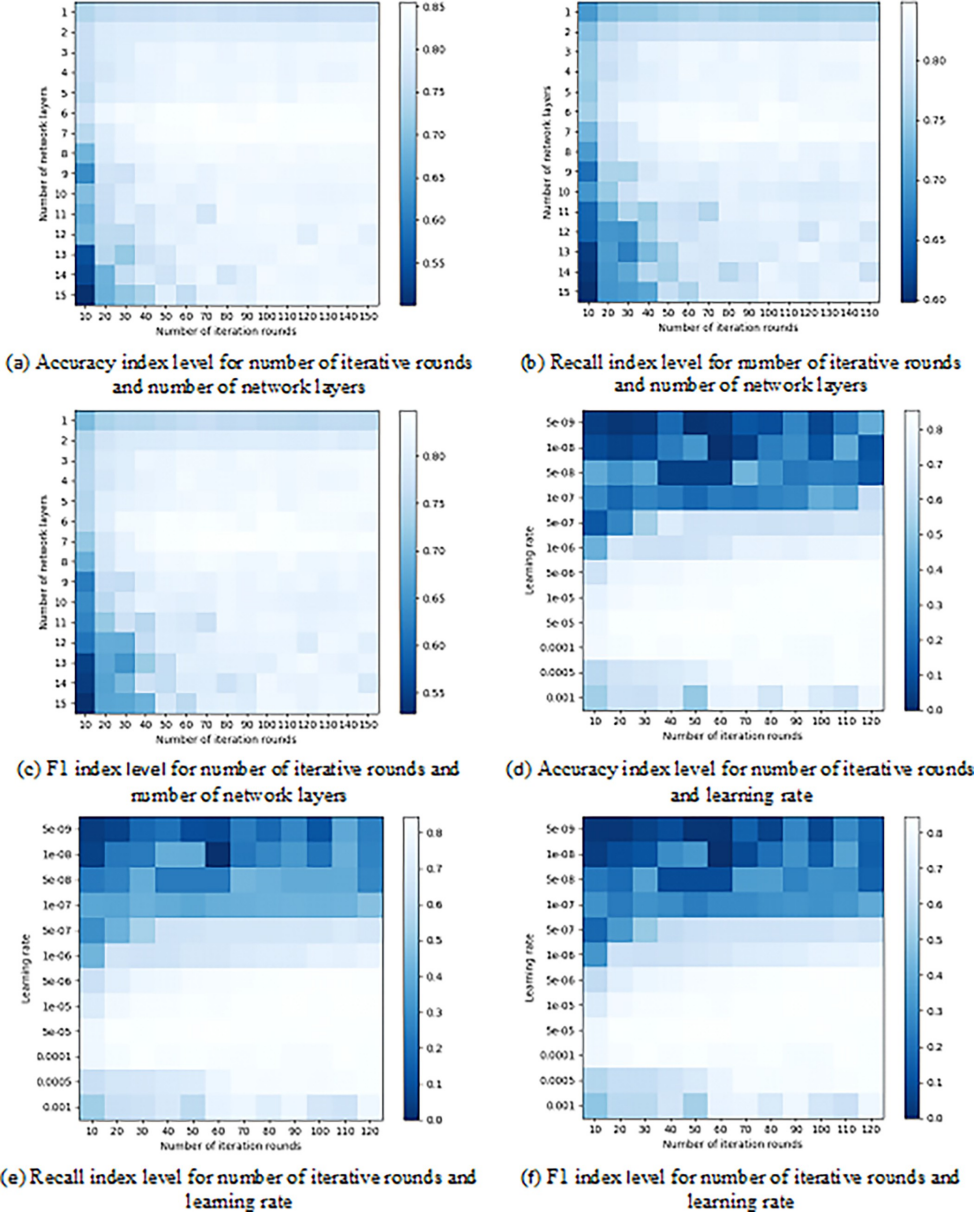
Performance level of PTM-MFGCN with different hyperparameters. (a) Accuracy index level for number of iterative rounds and number of network layers. (b) Recall index level for number of iterative rounds and number of network layers. (c) F1 index level for number of iterative rounds and number of network layers. (d) Accuracy index level for number of iterative rounds and learning rate. (e) Recall index level for number of iterative rounds and learning rate. (f) F1 index level for number of iterative rounds and learning rate.

To investigate the impact of hyperparameters on the performance of the PTM-MFGCN model, this study conducts a detailed analysis and comparison of several crucial hyperparameters, including the number of network layers, learning rate, and number of iterative rounds. The number of network layers is L ∈ {1, 5, 10, 15, 20, 25, 30, 35, 40, 45, 50, 55, 60, 65, 70}, the learning rate is R ∈ {5e-9, 1e-8, 5e-8, 1e-7, 5e-7, 1e-6, 5e-6, 1e-5, 5e-5, 0.0001, 0.0005, 0.001}, and the number of iterative rounds is E ∈ {10, 20, 30, 40, 50, 60, 70, 80, 90, 100, 110, 120, 130, 140, 150}. For the sake of experimental fairness, apart from the hyperparameters under current investigation, the remaining hyperparameters are set the same as in Section 5.1. The experimental results are illustrated in Fig 6.

Fig 6A to 6C gives the relationship between the number of network layers, the number of iterative rounds and the evaluation indexes. It can be seen that when the number of network layers of the model is 6 or 7, the overall performance of the model is good. When the number of network layers is 1 to 3, the model is underfitted and the overall performance of the model is poor. When the number of network layers of the model is 13 to 15, the model appears to be overfitted and the overall performance of the model decreases. The experimental results indicate that the number of iterative rounds and the number of network layers are sensitive parameters. When the number of iterative rounds and the number of network layers is too small, it may result in underfitting issues, wherein the model fails to adequately express the rich semantic information of all entity relationships. Conversely, when the number of iterative rounds and the number of network layers is too large, overfitting phenomena may arise, leading to a decline in performance. Fig 6D to 6F gives the relationship between learning rate, number of iterative rounds and evaluation indexes. It can be observed that the model performs well when the number of iterative rounds from 10 to 60, and the learning rate is within the range of 1e-5 to 0.001. The model exhibits good performance when the number of iterative rounds from 70 to 120, and the learning rate is within the range of 5e-6 to 0.005. As the number of iterative rounds or the learning rate increases, the evaluation indexes of the model first tend to increase, then stabilise, and then start to slowly decrease. The experimental results similarly demonstrate that the learning rate and the number of iterative rounds are sensitive parameters, and their excessively small or large values may give rise to the problems of underfitting or overfitting in the model.

### Ablation experiments

In order to assess the impact of pre-training tasks utilizing term masking and multi-feature graph convolution on model performance. In this study, two variant models, namely PTM-MFGCN-CUT and PTM-MFGCN-UNI, were designed based on the PTM-MFGCN model. PTM-MFGCN-CUT denotes the removal of term masking pre-training from the PTM-MFGCN model, while PTM-MFGCN-UNI signifies the removal of multi-feature graph convolution functionality. The PTM-MFGCN model and its variant models demonstrate the average precision, recall, and F1 score in entity relation extraction task, as presented in Table 4.

**Table 4:** Comparison of indexes between PTM-MFGCN and variant models.

Models	*P*	*R*	*F1*
**PTM-MFGCN-CUT**	59.74	76.98	67.23
**PTM-MFGCN-UNI**	84.75	82.14	81.53
**PTM-MFGCN**	89.92	90.81	90.27

From Table 3, it can be observed that PTM-MFGCN-CUT achieves precision, recall, and F1 scores of 59.74, 76.98, and 67.23, respectively. Similarly, PTM-MFGCN-UNI attains precision, recall, and F1 scores of 84.75, 82.14, and 81.53, while PTM-MFGCN demonstrates precision, recall, and F1 scores of 89.92, 90.81, and 90.27, respectively. Compared to the PTM-MFGCN-CUT model, PTM-MFGCN demonstrates a remarkable increase of 50.52% in precision, 17.97% in recall, and 34.27% in F1 score. Due to the removal of term masking pre-training in the PTM-MFGCN-CUT model, its ability to extract specialized terminology in emergency plans has diminished, resulting in a decline in model performance. Compared to PTM-MFGCN-UNI, PTM-MFGCN achieves a improvement of 6.10% in precision, 10.56% in recall, and 10.72% in F1 score. PTM-MFGCN-UNI, which adopts a single-feature strategy, exhibits a weakened ability to extract complex relationships, resulting in an overall decline in model performance.

### Case retrieval experiments for emergency knowledge graphs

**Fig 7:**
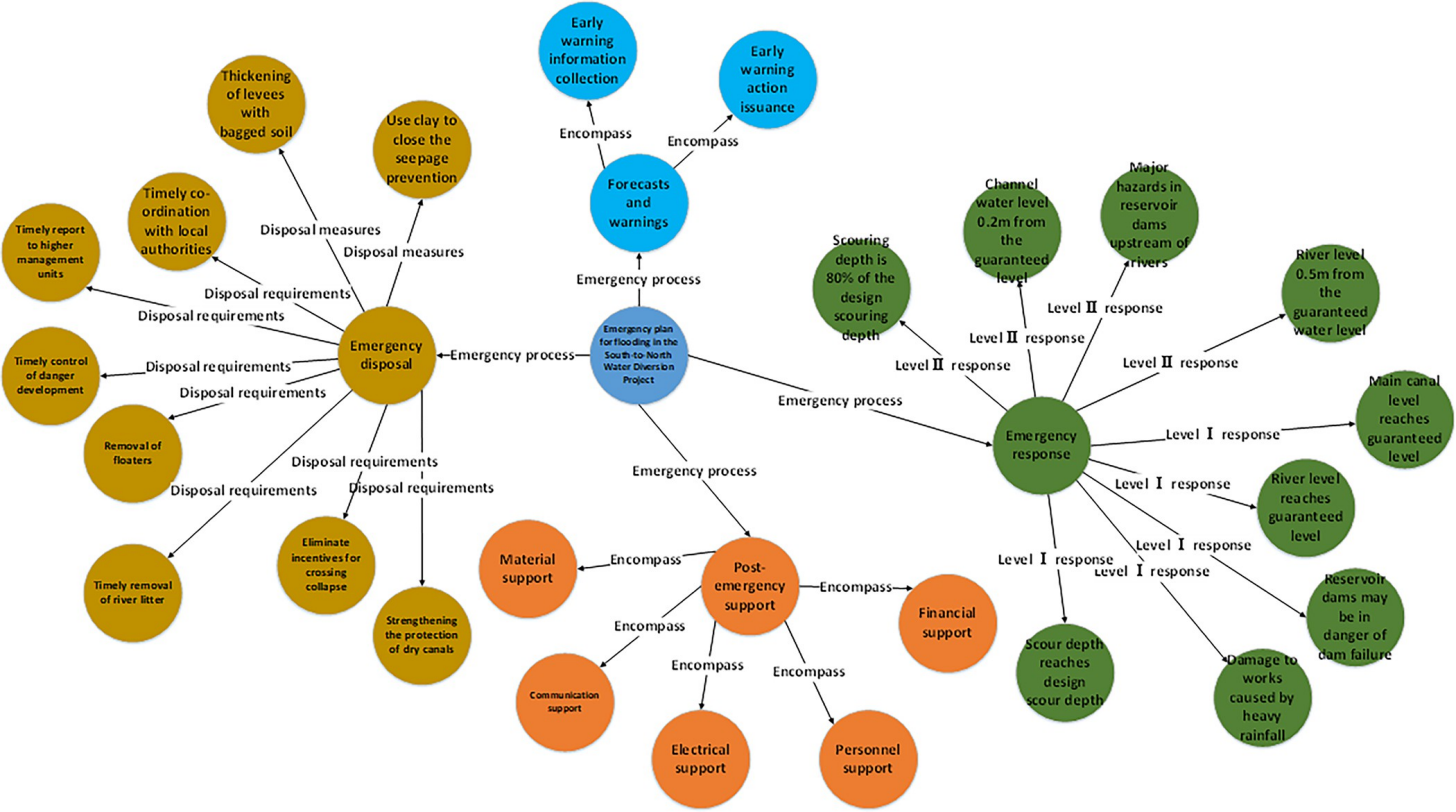
Display of emergency knowledge graph case retrieval results.

Taking " Emergency process of flooding in South-to-North Water Diversion Project" as an example, the results of searching the emergency knowledge graph are shown in Table 5 and Fig 7. The emergency process of flooding in the South-to-North Water Diversion Project is divided into forecast and early warning, graded response, emergency disposal and post-security. Among them, forecasting and warning contains knowledge related to the collection and release of warning information. The graded response contains Ⅰ ~ Ⅳ level of emergency response standards. Emergency disposal includes disposal measures and disposal requirements. Post-security includes team security, communication security and power security. According to the information presented in Table 5 and Fig 7, the flood emergency process is basically included in the list of returned results, and the usability of the knowledge is high. The current search results combined with the actual situation of the South-to-North Water Diversion Project can basically meet the demand for accurate push of emergency knowledge. For the small number of correct content not covered in the recommendation list, the main reasons for recommendation failure are as follows. ① Due to the possibility of annotation errors during manual supervision of data labeling, the entity recognition algorithm may encounter difficulties in accurately identifying certain entities. ② The insufficiency of training data for the entity recognition model has led to an inability to accurately identify pertinent entities.

**Table 5:** Emergency knowledge graph case retrieval related entities and attribute values.

Search content	Associated entities	Associated attributes
Forecast and warning	Early warning information collection Early warning information dissemination	Evaluation of engineering safety Realise information sharing among departments Timely reporting of engineering safety risks
Grading response	Ⅰ response standard Ⅱ response standard Ⅲ response standard IV response standard	Scour depth up to the calculated scour depth Damage to engineering structures due to flooding Possible dam failure of reservoirs Water level reaches guaranteed level
Emergency disposal	Disposal measures Disposal requirements	Emergency dispatch of channels Timely coordination with local governments Timely report to the higher management unit
Post-Security	Team security Communication security Electrical security	Organisation of emergency protection teams Establishment of rescue co-operation mechanism Inspection of network communications Inspection of power supply lines

## Conclusion

1. In this study, a pre-training task based on terminology masking is proposed to address the problem of numerous terminologies in water diversion project emergency plan texts. Compared to BERT-BILSTM-CRF, PTM-MFGCN exhibits improvements in accuracy, recall, and F1 score, with an increase of 1.24%, 2.54%, and 1.97% respectively. This enables it to effectively perform the task of extracting emergency entities in water diversion projects.
2. To address the issue of complex relationships in emergency plan texts, a relationship extraction method based on multi-feature graph convolution has been devised. The experimental results demonstrate that PTM-MFGCN achieves an accuracy, recall, and F1 score of 85.18%, 84.33%, and 84.60%, respectively. The model exhibits superior overall performance, showcasing its robust capability in handling complex graph-structured data.
3. The extracted triplets are stored using the Neo4j graph database, creating an emergency knowledge graph in the field of water diversion projects. By querying the knowledge graph, the semantic relationships between emergency knowledge can be clearly displayed.
4. The current endeavor still possesses ample room for improvement, and forthcoming efforts will be directed towards the investigation of the multimodal challenges present in the textual documentation of water diversion project emergency plan.

## Supporting information

S1 File (DOCX)

S2 File (DOCX)
