# Supplementary material for: Emergency entity relationship extraction for water diversion project based on pre-trained model and multi-featured graph convolutional network
Source: PLoS One. 2023 Oct 9;18(10):e0292004. doi: 10.1371/journal.pone.0292004 (PMC10561837; doi:10.1371/journal.pone.0292004)
Supplement: S1 File — (DOCX) [file pone.0292004.s001.docx]

All relevant source code for the software is uploaded to the public code repository GitHub, accessible at <https://github.com/LIHU-WANG/PTM_MFGCN>
