# Supplementary material for: Emergency entity relationship extraction for water diversion project based on pre-trained model and multi-featured graph convolutional network
Source: PLoS One. 2023 Oct 9;18(10):e0292004. doi: 10.1371/journal.pone.0292004 (PMC10561837; doi:10.1371/journal.pone.0292004)
Supplement: S2 File — (DOCX) [file pone.0292004.s002.docx]

**List of symbol definitions**

| Abbreviations | Full name | Abbreviations | Full name |
| --- | --- | --- | --- |
| PTM-MFGCN | multi-feature graph convolutional network based on pre-trained model | RNN | Recurrent neural networks |
| Att-BILSTM | bidirectional long short-term memory network model based on attention mechanism | CRF | Conditional random field |
| BIGRU | bidirectional gated recurrent unit network | BIGRU-CRF | integration of BIGRU and CRF |
| CNN | convolutional neural networks | GNN | graph neural networks |
| GCN | graph convolutional networks | BERT | bidirectional encoder representation from transformers |
| GPT | generative pre-trained transformer | T5 | text-to-text transfer transformer |
| BERT-CNN | integration of BERT and CNN | BERT-GCN | integration of BERT and GCN |
| GRN | graph recursive neural network | GAT | graph attention network |
| BERT-BILSTM-CRF | integration of BERT, BILSTM and CRF |  |  |
| Variables | Meaning | Variables | Meaning |
| $S_{p}$ | concatenated vector matrix of part-of-speech | $p_{k}$ | vector representation of the $k$th word in the text |
| $S_{q}$ | character vector matrix | $q_{k}$ | vector representation of the $k$th character in the sample |
| $f$ | classification function | $W$ | weight matrix |
| $b$ | bias term | $\bigoplus$ | concatenation operator |
| $A\epsilon R^{n*n}$ | adjacency matrix | $A^{psc}$ | part-of-speech combination vector |
| $A^{sdt}$ | syntactic dependency vector | $A^{tbd}$ | tree-based distance vector |
| $A^{rpd}$ | position vector | $S$ | text sequence |
| $[MASK]$ | concealed term characters | $E_{[MASK]}$ | masked term entities |
| $p_{[MASK]}$ | masked term context | $X$ | word vectors extracted using the pre-training model |
| $\hat{y}^{(i)}$ | output of the model | $y^{(i)}$ | true labels |
| $T_{P}$ | number of correctly predicted entities | $N$ | number of entity triplets |
| $F_{N}$ | number of entities that were not predicted | $F_{P}$ | number of predicted entities that are not actual entities |
